# A ROM-Less Direct Digital Frequency Synthesizer Based on Hybrid Polynomial Approximation

**DOI:** 10.1155/2014/812576

**Published:** 2014-04-23

**Authors:** Qahtan Khalaf Omran, Mohammad Tariqul Islam, Norbahiah Misran, Mohammad Rashed Iqbal Faruque

**Affiliations:** ^1^Department of Electrical, Electronic and Systems Engineering, Faculty of Engineering & Built Environment, Universiti Kebangsaan Malaysia (UKM), 43600 Bangi, Malaysia; ^2^Institute of Space Science (ANGKASA), Faculty of Engineering & Built Environment Building, Universiti Kebangsaan Malaysia (UKM), 43600 Bangi, Malaysia

## Abstract

In this paper, a novel design approach for a phase to sinusoid amplitude converter (PSAC) has been investigated. Two segments have been used to approximate the first sine quadrant. A first linear segment is used to fit the region near the zero point, while a second fourth-order parabolic segment is used to approximate the rest of the sine curve. The phase sample, where the polynomial changed, was chosen in such a way as to achieve the maximum spurious free dynamic range (SFDR). The invented direct digital frequency synthesizer (DDFS) has been encoded in VHDL and post simulation was carried out. The synthesized architecture exhibits a promising result of 90 dBc SFDR. The targeted structure is expected to show advantages for perceptible reduction of hardware resources and power consumption as well as high clock speeds.

## 1. Introduction

Recent applications of digital communication impose rigid specifications on frequency synthesizers, which include the ability to achieve ultrathin frequency increments, low spurs level, and fast switching speed, with an efficient power system. Direct digital frequency synthesizers (DDFS), among other frequency synthesizer types, exhibit a greater flexible capability to satisfy these needs, which are rapidly growing.

**Figure 1:**
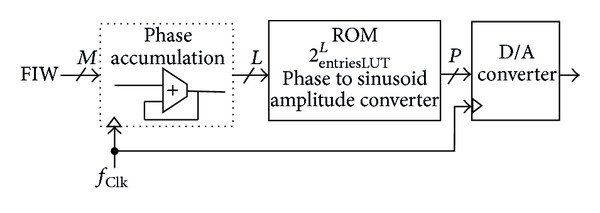
The basic structure of a DDFS.

A classical DDFS architecture, introduced by Tierney et al. [1] and shown in Figure 1, is no longer used. Extensive research efforts, during the last four decades, have led to major modifications in Tierney's architecture, even to the extent of introducing alternative architectures that no longer employ the concept of a lookup table (LUT). The aim is to imitate the proficiency of ROM-based DDFS in terms of signal integrity, using the lowest computational cost.

One of the most interesting concepts that have been explored is based on polynomial approximation, in which the actual input phase sample is fed directly into an algorithm to construct the sine curve, rather than indexing the power-hungry bulky ROM.

Following this concept, ROM-less DDFS have been reported based on a special phase conversion algorithm such as a Taylors series evaluation as in [2], a coordinate rotation digital computer (CORDIC) algorithm as in [3], a first-order Chebyshev approximation as in [4, 5], a piecewise first-order polynomial approximation as in [6, 7], a second-order parabolic approximation as in [8], a two-segment fourth-order parabolic approximation [9], and an eighth-order even polynomial as in [10], among others. As reported, using a high-order polynomial approximation, high SFDR can be achieved, which is highly desirable. In contrast, the high switching speed required by recent applications in wireless communication is difficult to obtain using any method that is based on high-order polynomials [11].

As the polynomial's order increases, the number of polynomial coefficients increases, resulting in extra arithmetic operations. The additional circuits that are needed occupy a large amount of die area, consuming significant power and slowing down the whole system's speed. To circumvent this problem, segmentation, with an adequate polynomial approximation for each segment interval, appears to be a perfect solution. In this case, the appropriate polynomial approximation can be individually and nicely fitted to each sine curve segment. Therefore, in this paper, we introduce a proposed DDFS architecture design, which is based on a hybrid polynomial approximation. It is expected that this new combination can precisely approximate the sine curve, requiring only minimal segmentation, thereby achieving excellent spectral purity with lower employed circuitry and working at high clock rates with low power consumption. This paper is structured as follows.  Section 2 describes the proposed DDFS algorithm. The efficient proper polynomial arrangements are discussed in Section 3. Polynomial coefficient digitization is analyzed in Section 4. DDFS architecture design including radian phase accumulator, constant coefficient multipliers, squarer and variable coefficient multiplier architectures are explained in detail in Section 5.  Section 6 describes the gate level simulation results while Section 7 presents the experimental findings, and Section 8 concludes the paper.

## 2. Proposed DDFS Algorithm 

For a sine curve approximation, it is desirable to partition the first quadrant interval [0–*π*/2] into small subintervals and then approximate each interval by polynomials of a certain degree, rather than employing a single polynomial approximation. It goes without saying that the more divided the interval, the more accurate the resulting approximation will be, and it is also true that the higher the order, the better the approximation that is achieved. However, increasing the number of sub-intervals will stretch the coefficients' ROM, and raising the polynomial degree will increase the number of polynomial coefficients, resulting in extra arithmetic circuitry being required. This study exploits the nature of the sine curve, where the portion near the zero point can be represented by a single linear segment, and the remaining part is represented by another segment with an adequate degree polynomial. As a result, the approximation employs the least number of segments and with a lower polynomial order. The process should be subject to balancing the enhancements of spectral purity against the hardware complexity.

**Figure 2:**
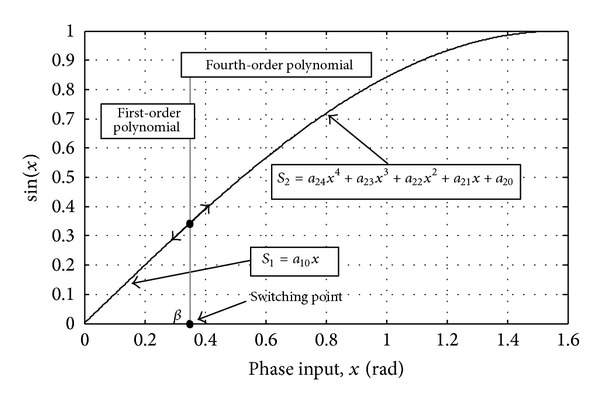
The principle of hybrid polynomial approximation.

Figure 2 shows this principle where the phase sample *β* (the polynomial type switching phase point) can be anywhere on the phase input axis from zero to *π*/2. A fourth-order polynomial has been chosen to approximate the second segment, which represents a compromise solution to face the tradeoff between a high degree polynomial approximation and digital implementation requirements. The approximated function can be expressed as follows:

(1) $$\begin{array}{c} f\left(x\right)=\{\begin{array}{cc} a_{10}x & 0\leq x\leq \beta ,\\ a_{24}x^{4}+a_{23}x^{3}+a_{22}x^{2}+a_{21}x+a_{20}, & \beta \leq x\leq \frac{\pi }{2}, \end{array} \end{array}$$

where *a*_*n*,*i*_ (*n* = 1, 2, *i* = 0, 1, 2, 3, 4) represents the coefficients of the polynomials and *β* is the linear segment upper bound. The extreme points *β* = 0 and *β* = *π*/2 represent a single fourth- and single first-order polynomial approximation, respectively.

Next, we have to determine the appropriate value of *β* that corresponds to the maximum SFDR, and to do so, we have to first find the optimal sets of polynomial coefficients for certain points *β* ∈ [0, *π*/2], and then, for each set of real-valued coefficients, we have to determine the corresponding SFDR level. For this purpose, we employed a powerful MAPLE optimization package to apply the following Minimum-Mean Square Error (MMSE) criterion:

(2) $$\begin{array}{c} \text{MMSE}=\underset{\left[a_{ni,n=1,2,i(0\cdots 4)}\right]}{\min}\overset{\pi /2}{\underset{0}{\int }}{\left[\sin(x)-f(x)\right]}^{2}\;dx. \end{array}$$

**Figure 3:**
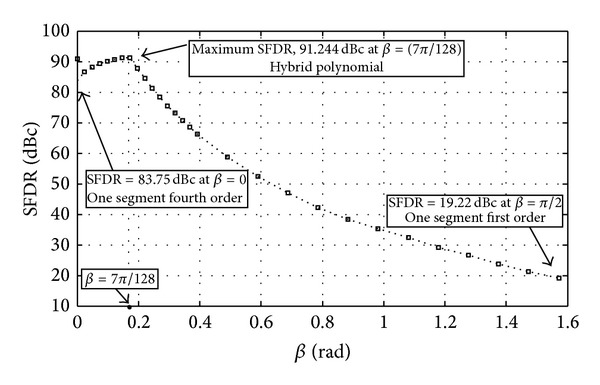
SFDR upper bounds of the hybrid polynomial approximation versus *β*.

The values of SFDR are found with the aid of MATLAB and are depicted in Figure 3. Next, we examine the behavior of the approximated function with respect to *β*. The aim is to Figure out the maximum achievable SFDR. From the plot, we observe that for *β* = 0, the *f*(*x*) is minimized to a single fourth polynomial approximation, *f*(*x*) = *a*_24_ *x* ^4^ + *a*_23_ *x* ^3^ + *a*_22_ *x* ^2^ + *a*_21_ *x* + *a*_20_, 0 ≤ *x* ≤ *π*/2,and the approximated function has an SFDR of 83.75 dBc. During the 0 ≤ *β* ≤ 7*π*/128 interval, the SFDR gradually increases until it reaches its maximum level of 91.244 dBc at *β* = 7*π*/128.

This result is in line with the expected result. The value of sin(*x*) is almost equal to *x* during this interval, and therefore the linear segment has been fitted precisely to the sine curve. Beyond this point, the SFDR is decreased until reaching the lowest level at *β* = *π*/2. The approximated function becomes *f*(*x*) = *a*_10_ *x*, 0 ≤ *x* ≤ *π*/2, which represents a single first-order polynomial approximation. Substituting the phase sample point *β* = 7*π*/128 in (1) yields

(3) f(x)={a10x  0≤x≤7π128,a24x4+a23x3+a22x2+a21x+a20,7π128≤x≤π2.

The optimal set of polynomial coefficients *a*_*ni*_ is obtained and is presented in Table 1.

**Figure 4:**
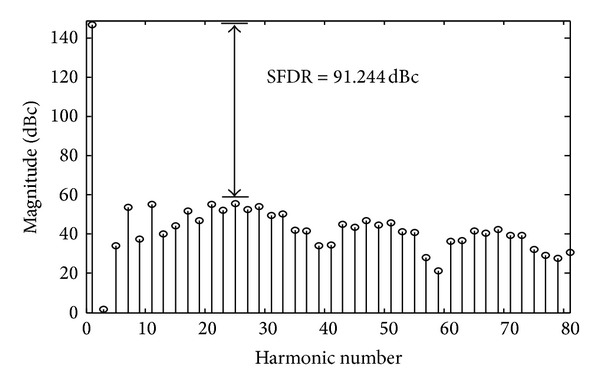
Hybrid polynomial SFDR with nondigitized optimal coefficients.

**Figure 5:**
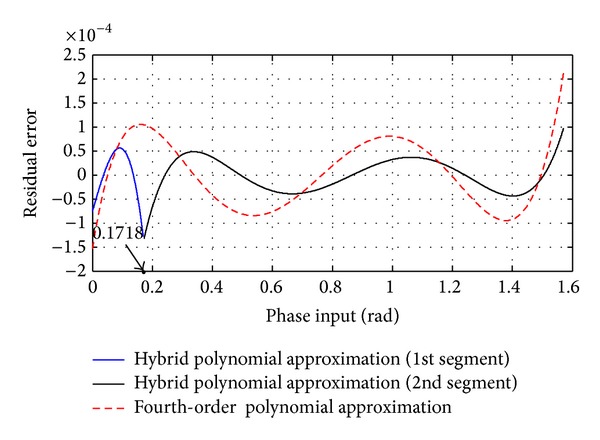
The residual error of the approximated sinusoidal wave.

Figure 4 shows the spectrum of the *f*(*x*) for this set of real-valued coefficients. The largest unwanted frequency component has an amplitude of −91.244 dB with respect to the target sinusoid and is also indicated.  Figure 5 shows the residual error of the approximated sinusoidal wave. The maximum absolute error (MAE) is equal to 1.25 × 10^−4^ (0.000125 < 2^−12^).

To show the contribution of the linear segment, the residual error of the approximated sine curve, based on one segment fourth-order approximation, is also shown (dashed red line), with MAE equals 2.2 × 10^−4^. Conspicuously, the residual error is observed to be much lower for the same polynomial approximation when it is combined with the linear segment.

## 3. Efficient Polynomial Arrangement 

Before quantizing the polynomial coefficients, we have to simplify the approximated function. The aim is to produce the targeted sine output with a minimum of arithmetic evaluation. The hard part is the fourth-order polynomial evaluation, which requires more careful handling. Many arrangements have been proposed to simplify the computation of high-order polynomials [9, 10]. Most of these embody the Horner form. Forming a polynomial in the Horner arrangement results in efficient computation, and for this reason, the Horner arrangement has been extensively used in high-order polynomial-based sine approximations. Thus, the usual way to simplify this type of polynomial is to use a nested multiplication algorithm (NMA) as follows:

(4) $$\begin{array}{c} f\left(x\right)=a_{20}+x\left(a_{21}+x\left(a_{22}+x\left(a_{23}+a_{24}x\right)\right)\right). \end{array}$$

Evaluation of such a polynomial requires four multipliers, three of which have variable operands that cannot be simplified. One of the most interesting techniques for simplifying the evaluation of the variable coefficient multiplier is to replace it with its squarer counterpart. Following this idea, [9] employed the following arrangement:

(5) $$\begin{array}{c} f\left(x\right)={\left({\left(ax+b\right)}^{2}+c\right)}^{2}+\delta ,\;\delta =k_{0}x+k_{1}. \end{array}$$

In this case, the implementation requirement was reduced to two squares and two constant multipliers. Accordingly, the hardware intricacy was significantly reduced. Now, in our design and for further simplification, we choose to remain close to the ideas of replacing the variable multiplier and using the nested form. To do so, we apply the arrangement presented in [14]. Press et al. [14] stated that a polynomial of order *n* greater than 3 can be determined using less than *n* multiplications if some auxiliary coefficients are assumed to be computed in advance, usually with a penalty of doing an extra addition. Following [14] for a Quartic polynomial, *P*(*x*) = *a*_0_ + *a*_1_ *x* + *a*_2_ *x* ^2^ + *a*_3_ *x* ^3^ + *a*_4_ *x* ^4^. This can be evaluated using three multiplications and five additions as follows:

(6) $$\begin{array}{c} P\left(x\right)=\left[{\left(c_{1}x+c_{2}\right)}^{2}+c_{3}\right]\left[{\left(c_{1}x+c_{2}\right)}^{2}+c_{1}x+c_{4}\right]+c_{5}, \end{array}$$

where the *c*_*i*_, *i* = 1, 2, 3, 4, 5, represents the new polynomial coefficient. Using this arrangement, we can easily deduce the new coefficients of (6) from the standard arrangement and Table 1. The resulting values are reported in Table 2.

According to (6), the implementation cost is reduced to one squarer, one constant multiplier, and one variable operand multiplier.  Table 3 summarizes the required arithmetic circuits for the proposed arrangement in comparison to the prior reported arrangements.

## 4. Polynomial Coefficients Digitization 

To complete the design, in the following we will quantize the optimal real-valued coefficients (detailed in Table 2) as well as the coefficient of the linear segment *a*_1,0_ presented in Table 1. Reducing the coefficient word length is highly desirable: the lower the coefficient's word length, the lower the hardware computational cost. In contrast, excessive quantization may further decrease the SFDR level. The design has to balance circuit complexity against quantization accuracy [15].

To satisfy the targeted SFDR level, the coefficient detailed in Table 2 is quantized with sufficient finite precision as follows:

(7) $$\begin{array}{c} c_{iq}=\frac{\left\lfloor 2^{N}c_{i}+0.5\right\rfloor }{2^{N}},\;i=1,2,3,4,5. \end{array}$$

Where ⌊·⌋ denotes the floor function, *N* is the coefficient word length, and 0.5 ensures that the halfway values (2^*N*^ *c*_*i*_) are rounded up. The resulting coefficients are shown in Table 4. The coefficients *c*_1_, *c*_2_, *c*_3_, and *c*_4_ are quantized to 13 bits, and the coefficient *c*_5_ is quantized to 14 bits. The coefficient *a*_1,0_ is quantized to 10 bits. The phase boundary value (*π*/2) is quantized to 13 bits.

**Figure 6:**
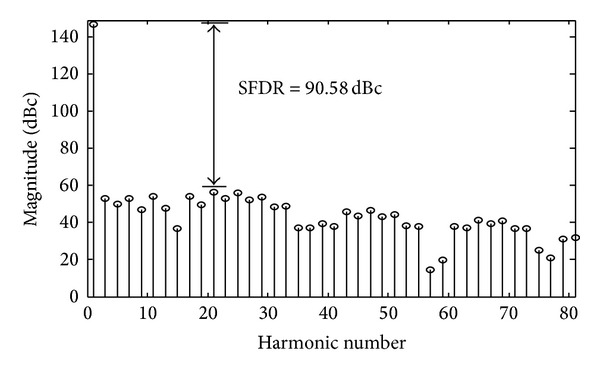
Hybrid polynomial SFDR with digitized optimal coefficients.

**Figure 7:**
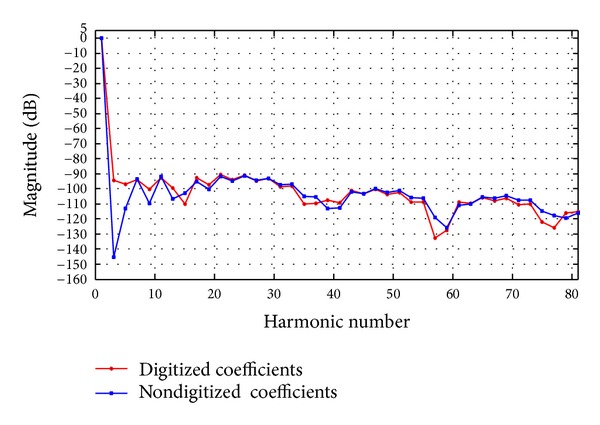
SFDR level for nondigitized and digitized optimal coefficients.

Next, we have to analyse the spurs level in the presence of quantization error.  Figure 6 shows the resulting spectrum where the largest unwanted frequency component has an amplitude of −90.58 dBc, and for comparison purposes, the spurs distributions of the digitized and nondigitized coefficients have been depicted together in Figure 7.

## 5. DDFS Architecture Design

**Figure 8:**
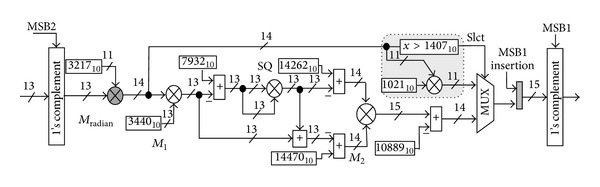
Architecture of the proposed algorithm. *M*_radian_: radian multiplier, *M*_1_: constant coefficient multiplier, *M*_2_: variable coefficient multiplier, *SQ*: squarer, MSB1: first most significant bit, and MSB2: second most significant bit.

The schematic of the sine generator based on the hybrid polynomial approximation is shown in Figure 8. The proposed architecture is composed of mainly two parts, the linear part (the shaded rectangle), which has one constant multiplier, and the fourth-order polynomial part, which has one constant multiplier, one squarer, one variable coefficient multiplier, and five adders.

For proper operation, one part must be selected at a time. For this purpose, a multiplexer is used to switch between the two polynomials at a specific phase sample. Furthermore, the architecture employs the normalized phase accumulator (PA) in conjunction with a simple constant coefficient multiplier instead of the complex modulo *π*/2 phase accumulator. The issue of choosing the appropriate phase accumulator is discussed further in the following section.

### 5.1. Radian Phase Accumulator

In the LUT-based phase-to-sine amplitude converter (PSAC), the phase accumulator is a simple *M*-bit binary adder, followed by a clocked phase register. At each clock cycle, the phase accumulator updates its phase register with a new phase sample simply by incrementing the previous output by an amount FIW, the frequency instruction word, which is a unique, predefined binary number for a certain output frequency. An overflow occurs whenever the sum of the adder operands exceeds its capacity (2^*M*−1^) coincident to one period of the synthesized waveform. The resulting phase sample is then used for indexing the sine-amplitude lookup table (LUT). However, for our case, the aforementioned phase accumulator architecture is not applicable and a more complicated modification is required. The contents of the phase register represent the input phase [0, *π*/2], normalized by *π*/2 in the interval [0, 1], whereas the 4th-order approximation defined in the above equations needs to evaluate the sine amplitude sample from the actual input phase sample measured in radians. Therefore, the normalized phase sample needs to be mapped into an equivalent radian value in the interval [0, *π*/2] before being applied to the input of the sine generator.

To the best of our knowledge, there are two approaches that have been proposed to convert the normalized phase accumulator to the radian counterpart. The first approach used a modulo *π*/2 arithmetic. In this case, the *M*-bit phase accumulator needs to be truncated at the nearest integer to (*π*/2) · 2^*M*−2^ = *π* · 2^*M*−3^, and for generation the second quadrant, the common simple negation circuit has to be replaced by (*π* · 2^*M*−3^ − *θn*) two's complement adder and multiplexer, where *θn* = *n* × *FIW* represents the instantaneous accumulated phase sample. An extra gate for controlling the sine symmetry is also required. This technique was used in [4, 9, 10] but suffers from amplitude mismatching between successive quadrants at extreme points (0, *π*/2). In other words, as the step increment FIW is any quantity in the range of FIW [2^*M*−1^ : 1], the truncated point will then be (*π* · 2 ^*M*−3^ − (FIW mod⁡ *π* · 2^*M*−3^)), 0 ≤ (FIW mod⁡ *π* · 2^ ^^*M*−3^) < FIW. The next quadrant (as designed) starts, this time, from *π* · 2^*M*−3^ not from (*π* · 2^*M*−3^ − (FIW mod⁡ *π* · 2^*M*−3^)), and, therefore, an amplitude discontinuity of (FIW mod⁡ *π* · 2^*M*−3^) occurs.

The second approach uses the normalized phase accumulator and hence multiplies its phase output by a quantized *π*/2 constant coefficient. A similar hardwire multiplier has been used in CORDIC-based DDS architectures as a radian converter, as presented in [3].

By using the standard PA, the algorithm is free from the amplitude discontinuity and the controlling of quadrant symmetry is quite simple. At first glance, the alternative solution may seem slightly costly due to the added constant coefficient multiplier, but in the following section, we show that this can be performed with a simple arrangement, which reduces its required hardware resources significantly.

### 5.2. Constant Coefficient Multipliers

The complexity of the architecture, as is seen in Figure 8, is heavily dominated by the complexity of the squarer and variable multiplier circuits. The other constant coefficient multipliers are rather simple and they can be significantly simplified as follows.

**Figure 9:**
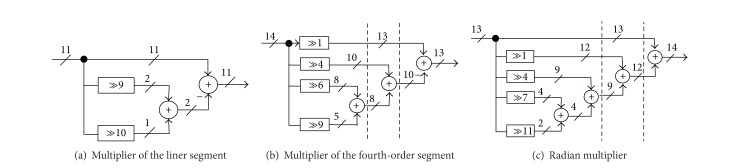
Constant coefficient multipliers.

For the linear part, which has one constant multiplier, we can apply the concept introduced by [13], based on which the coefficient can be expressed using the following canonic signed digit (CSD) representation:

(8) $$\begin{array}{c} a_{n}=\overset{m}{\underset{i=0}{\sum }}w_{i}2^{k}, \end{array}$$

where *w*_*i*_ ∈ {−1, + 1}, *k* ∈ *Z*, *Z* denote the set of all integers, and *m* is a fixed number, which has to be as small as possible for efficient realization. By doing this, the digital multiplier can be realized by summing the hardwired shifted versions of phase sample *x*. Following this concept, with 15-bit phase resolution, the coefficient *a*_10_ can be approximated by nine nonzero digits: 0.9970862 ≈ 1021/2^10^ = 2^−1^ + 2^−2^ + 2^−3^ + 2^−4^ + 2^−5^ + 2^−6^ + 2^−7^ + 2^−8^ + 2^−10^. The implementation of such a multiplier requires nine partial products, which need further simplification to be applicable. Instead, applying the analogy of Booth's encoding can help to reduce the partial products substantially. By Booth's encoding, the binary number can be represented by the difference of two binary numbers, which is assumed to be rather efficient in hardware implementation. This is true as long as the binary string consists of more consecutive ones. Following this concept, the coefficient *a*_10_ can be expressed by only three nonzero digits: 0.9970862 ≈ 1021/2^10^ = 1 − (2^−9^ + 2^−10^). The error is less than (0.000016 < 2^−15^), which is acceptable. In this case, the multiplier can be replaced by a simple two-operand adder as shown in Figure 9(a). It should be noted that the required right hardwired shifting does not involve a digital gate. Furthermore, the range of phase is limited to 7*π*/128. Therefore, we can reduce the adder word length to 11 bits when the phase boundary value (*π*/2) is quantized to 14 bits. The same procedure can be applied to the coefficient *c*_1_: 0.419941 ≈ 3440/2^13^ = 2^−2^ + 2^−3^ + 2^−5^ + 2^−7^ + 2^−8^ + 2^−9^, which has six partial products, and with Booth's encoding, the partial products are reduced to four 2^−1^ − (2^−4^ + 2^−6^ + 2^−9^) as depicted in Figure 9(b). The resulting error is (0.000019125 < 2^−15^) which is satisfactory. Following the same procedure, the radian multiplier with a constant coefficient of 3217/2^−11^ = 1.57080078125 can be expressed by five nonzero digits: 1 + 2^−1^ + 2^−4^ + 2^−7^ + 2^−11^ as shown in Figure 9(c). The error due to the quantization process is less than (0.00000445 < 2^−17^), which is highly sufficient.

**Figure 10:**
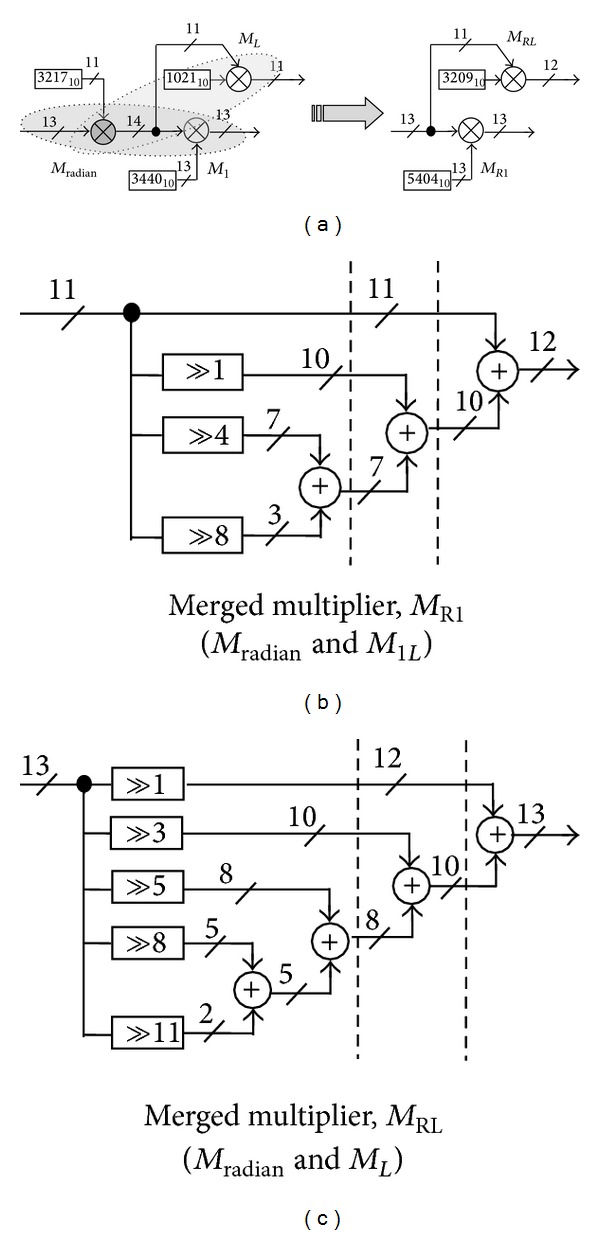
Alternative structures: the merged multipliers. *M*_radian_: radian multiplier, *M*_1_: constant coefficient multiplier, *M*_2_: variable coefficient multiplier, and *M*_*L*_: constant coefficient multiplier of the linear part.

Even though the constant multipliers are apparently in the simplest form, still another improvement can be achieved. The architecture displayed in Figure 8 can be further simplified by merely merging the cascaded multipliers in each path as depicted in Figure 10.

Here, *M*_*RL*_ = *M*_radian_ × *M*_*L*_ = 5404/2^13^ is the resultant merged multiplier of the radian and linear segment multipliers and *M*_*R*1_ = *M*_radian_ × *M*_1_ = 3209/2^11^ represents the merged multiplier of the radian and 4th-order segment multipliers. The reconstructed structure exhibits one multiplier less than the aforementioned arrangement.

### 5.3. Squarer Architecture

Next, we need to turn our attention to the design of the squarer and the variable multipliers, which represent the main sources of computational cost. For our design, it is important to keep the internal data path word length as small as possible to accommodate the data input word length for the subsequent arithmetic operations.

As shown in Figure 8, a 13-bit fixed-width squarer has been used instead of the regular 26-bit full-length squarer to satisfy the word length restriction. The simplest way to achieve such a fixed-width squarer is by omitting the less significant part at the partial product array (direct truncation) resulting in a significant area and decreased power consumption. For such truncation, a visible portion of useful information has normally been lost, resulting in high arithmetic error. Another type of truncation occurs at squarer output (post truncation). This type of truncation offers the best accurate fixed-width squarer [16], in which full partial products are realized, but the required hardware structure occupies significant die area. Next, one can think about reducing the partial products *PP* before applying the post truncation. In this case, only a small portion of *PP* needs to be realized, resulting in accurate fixed-width squarer with reasonable die area and power consumption. In designing the 13-bit squarer, following the above guideline, the most popular folding technique based on the symmetry of the partial products matrix has been used in conjunction with a Divide-and-Conquer approach.

We first divide the binary string input *X*_13−1_ into 2^6^ *X*_13−7_ & *X*_6−1_ partial components, so the squarer output can be approximated as follows:

(9) X13−12=(26X13−7+X6−1)2=212X13−72+2(26X13−7X6−1)+X6−12  ≈212X13−72+27X13−7X6−1.

The two terms of the last equation have common 7* LSB* zeros which can be truncated:

(10) $$\begin{array}{c} X_{13-1}^{2}\approx 2^{5}X_{13-7}^{2}+X_{13-7}X_{6-1}. \end{array}$$

**Figure 11:**
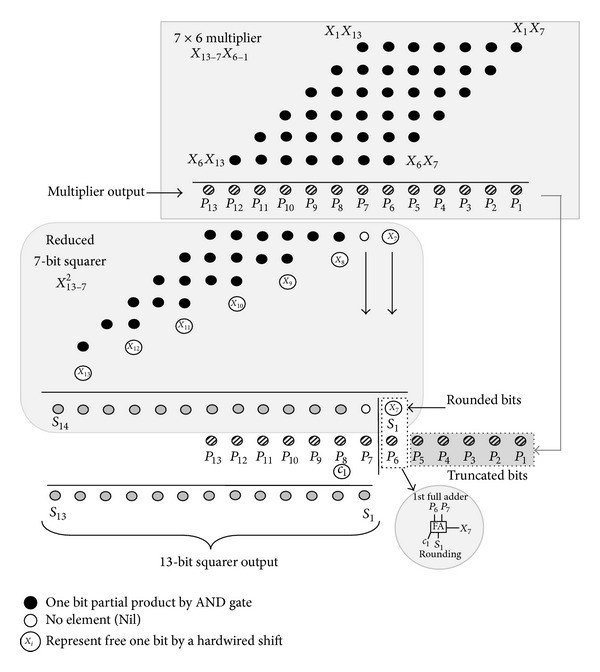
The reduced partial products matrix.

The first term in (10) represents the 7-bit primitive squarer, which can be heavily simplified by exploiting the symmetry property of the partial products matrix.  Figure 11 shows the reduced partial products matrix. The structure exhibits a partial products reduction of 50% in comparison with the standard multiplier. In an attempt to achieve an accurate fixed-width squarer, the 7 × 6 multiplier has been realized with full *PP*, which is also depicted in the same graph.

**Figure 12:**
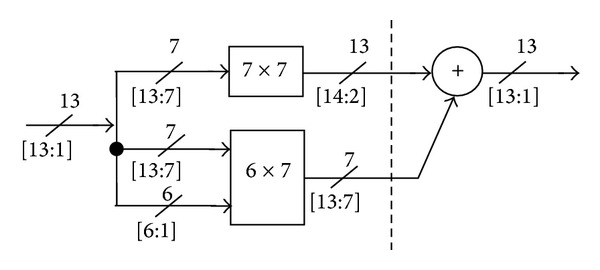
The 13-bit squarer circuit.

To satisfy the 13-bit fixed-width squarer, the 19-bit adder input has to be truncated. As a consequence, part of the realized *PP* needs to be truncated at the squarer output (post truncation). Accordingly, some die area and power consumption are normally wasted as a price for achieving high accuracy. One can instead apply a fixed-width multiplier, with the trade-off of high arithmetic error. Therefore, the given architecture in Figure 11 has been considered as a compromise solution in terms of accuracy and computational cost. A block diagram depicting the resulting 13-bit squarer is presented in Figure 12, with two pipelining stages.

### 5.4. Multiplier Architecture

As seen in Figure 8, with the 15-bit amplitude resolution required at the final stage, the 28-bit full-length multiplier output should be reduced to a 14-bit word length. However, by exploiting the fixed-width property, one can simplify the multiplier architecture, such that only the most significant *n* product bits are generated. Dropping the less significant partial products causes a substantial arithmetic error that has to be compensated for. Many error compensation methods for fixed-width multipliers have been proposed in [12, 16–18].

For our design, to implement the 14-bit multiplier, the fixed-width multiplier with linear compensation function introduced in [18] is employed. To do so, we first partition the *PP* matrix into MSP and LSP, where MSP and LSP are the most significant and the least significant parts, respectively. The LSP is then partitioned into LSP major and LSP minor subsets. Following [18], the LSP minor part is discarded, and an appropriate compensation function is then introduced to alleviate the impact of the dropped partial products.

## 6. Gate Simulation Results

**Figure 13:**
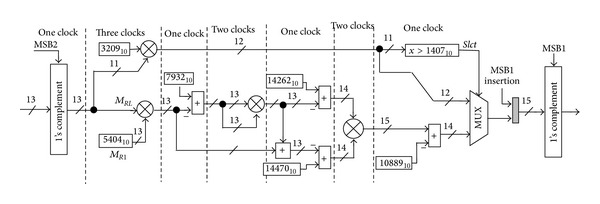
Pipelined version of the proposed architecture.

To validate the proposed algorithm, we have coded the design pipelined version architecture, seen in Figure 13, in VHDL using ALTERA QUARTUS II 12.1 software. The design included the arithmetic blocks shown in Figures 10 and 12 with 11 pipeline levels. The project synthesized with ALTERA Stratix IV FPGA (EP4SGX230KF40C2 device) and full compilation has been carried out.

**Figure 14:**
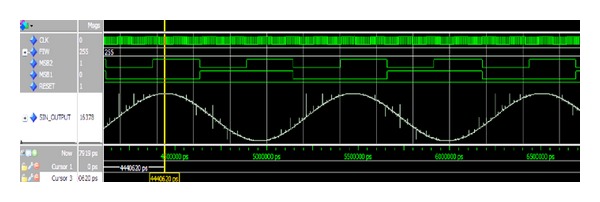
ModelSim Gate level simulation result.

**Figure 15:**
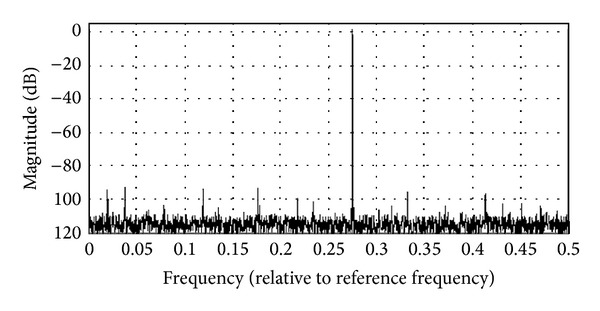
Calculated output spectrum.

The designed architecture was then analyzed with the ModelSim Altera 10.1b at both the Register Transfer Level (RTL) and Gate (Timing) level.  Figure 14 shows the synthesized output waveform observed by ModelSim with FIW = 255, amplitude resolution = 15 bits, and clock = 125 MHz. The data stream was then imported into MATLAB to evaluate the spurious level.  Figure 15 shows the output spectrum for an output clock frequency of 0.275, with FIW set to 9012. It is observed that an SFDR of 90 dBc is achieved as well.

## 7. Experimental Result and Comparison 

**Figure 16:**
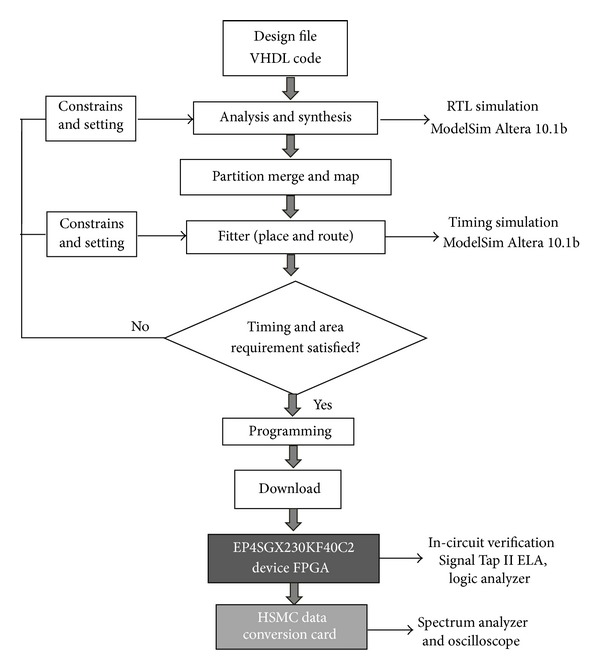
Design and debugging flowchart for FPGA-based DDFS implementation.

The next step to verifying the designed DDFS is by programming the targeted EP4SGX230KF40C2 device as shown in Figure 16. Note that the Stratix IV GX FPGA device is a part of the Altera DE4 development board. At this step, the Quartus II Programmer is activated to configure the EP4SGX230KF40C2 device. The generated project file is uploaded to the FPGA platform and the intended DDFS circuit is implemented in a physical FPGA chip. By this time, the functionality of a DDFS can be tested on a circuit board.

**Figure 17:**
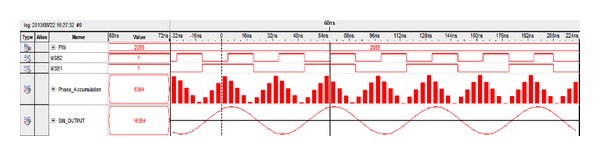
Signals Tap output waveform.

Instead of using external logic analyzer, we were using the powerful Signal Tap II embedded logic analyzer (ELA) to observe the output waveform. The Signal Tap II ELA is a system-level debugging tool integrated with Quartus II software capable of monitoring the real-time signal behavior in the FPGA design [19].  Figure 17 shows the synthesized output waveform of the DDFS with FIW = 2055, amplitude resolution = 15 bits, and clock = 125 MHz.

For further validation, the spurious level as well as the analog sine waveform can be observed experimentally using Rohde & Schwarz FSIQ3 Signal Analyzer and the Agilent DSO3202A Digital Storage Oscilloscope, respectively.

An available 14-bit digital to analog converter, DAC5672 from Texas Instruments, which is integrated with the Terasic AD/DA data conversion card, was used in this work [20]. The aforementioned High Speed Mezzanine Card (HSMC) can be add-on FPGA host board (Altera DE4 Development Board), where the targeted DDFS is implemented to convert the digital sine data stream into analogue waveform. Using the 14-bit unipolar DAC5672 imposes two modifications in our architecture as follows.

First, we have to modify the architecture to have a 14-bit amplitude resolution.

**Figure 18:**
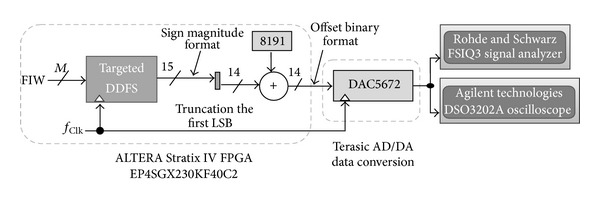
The proposed DDFS under test.

Second, we have to use the offset binary format instead of the two's complement for the digital data output. The system under test SUT is shown in Figure 18.

**Figure 19:**
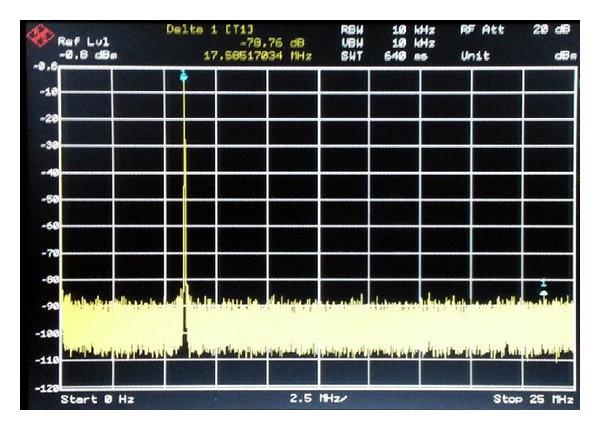
Spectrum of DDFS for FIW = 3998, DAC resolution = 14 bits, and clock frequency = 50 MHz.

**Figure 20:**
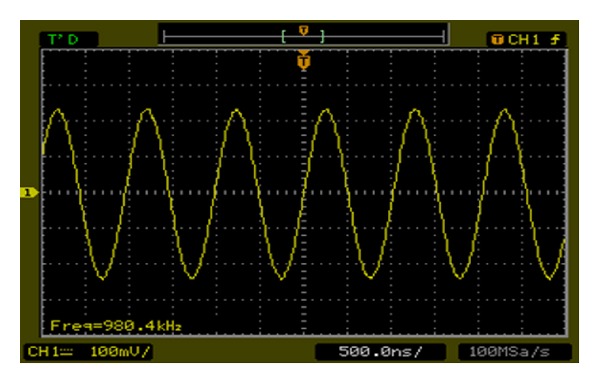
The output sine waveform of the implemented DDFS.

The output spectrum for the DDFS is shown in Figure 19 for *f*_out_ = 6.1 MHz and clock frequency = 50 MHz, which indicates spurious component of −78.7 dBc due to the 14-bit resolution of the DAC5672 used in this test. The analogue waveform is shown in Figure 20 for *f*_out_ = 0.9804 MHz.

The characteristics of the proposed work are summarized in Table 5 and compared with previously published algorithms. The power required has been estimated by the Power-Play Power Analyzer tool using a relative toggle rate of 25%.

As stated in the literature [13], it is difficult to achieve fair comparison between different DDFS circuits in terms of performances because of different implementation techniques, fabrication processes, frequency resolution, spurious level, and so on. One of the most interesting parameters that can aid in fair comparisons is normalized area. In the following, we introduce a simple method to obtain the approximate die area.

By using the Migration compatibility features in QUARTUS II 12.1 software, one can migrate the current FPGA device to the compatible Hard Copy IV ASIC device to find the equivalent 40-nm TSMC cells for the current FPGA logic utilization. In the same Quartus II project, the FPGA and a Hard Copy companion device have been designed using the FPGA first design flow.

We know that the Hard Copy IV has a 0.9 V core voltage using the 40-nm TSMC process, and each H-cell has 24-transistor cells [21]. For our device EP4SGX230KF40C2, the compatible Hard Copy is HC4GX35FF1517, and after full compilation, we found that the current designed project can fit within the targeted Hard Copy utilizing 938 H-cells, so the total number of employed transistors is 938 × 24 = 22512.

According to TSMC 40-nm technology [22], the static RAM cell size for a 40-nm process node is 0.242 *μ*m^2^, and each SRAM has 6 transistors. Thus, the equivalent SRAM can be found by dividing the total number of employed transistors by 6. The number of SRAM modules = 22512/6 = 3752, and the total area = 3752 × 0.242 *μ*m^2^ = 908 *μ*m^2^.

As illustrated in Table 5, if we exclude the work of [10], we can easily observe that the proposed work demonstrates the best performance in terms of area, power consumption, and speed. In comparison with the design present in [10], the DDFS in this paper also exhibits low power consumption, about one-twenty fourth that of architecture present in [10], and noticeable reduction in silicon area about one-fifth of a comparable [10] design, but it runs 0.86 lower speed, and shows 5 dBc SFDR less than the aforementioned architecture. One can indicate, for the best case, that the design from [10] exhibits little bit improvement in terms of speed and SFDR while it consumes much power and occupies large die area. However, in contrast, our design shows an excellent merit in almost all aspects.

To show the significance of the hybrid technique, a comparison with the work in [9] is helpful and important. The mentioned work used two segment fourth-order approximations, whereas this paper used one segment fourth-order and one segment first-order approximation. The comparison indicates that the proposed design exhibits the best performance in terms of area, switching speed, and power consumption while maintaining the same SFDR level of 90 dBc.

## 8. Conclusions

In this paper, we have presented a new DDFS architecture, using a combination of two carefully chosen polynomials to approximate the first sine quadrant. An exhaustive search was conducted to figure out the segment transition point that corresponds to the minimum approximation error. A simplified fourth-order polynomial architecture with low computational cost was introduced using only three multipliers. The squarer as well as the multiplier circuits were minimized, resulting in lower hardware implementation cost. The proposed DDFS was observed at the gate level. The spurious free dynamic range of a synthesized sinusoid achieved 90 dBc. The design was compared with an equivalent approach in terms of reduction of computation, speed, and power consumption. The comparison shows significant improvement in all features.

## Figures and Tables

**Table 1:** Optimal polynomial coefficient.

*n*	*a*_*n*,0_	*a*_*n*,1_	*a*_*n*,2_	*a*_*n*,3_	*a*_*n*,4_
1	0.9970862				
2	0.0011854	0.9894410	0.0330064	−0.2134780	0.0312845

**Table 2:** The resultant coefficients polynomial values.

*c*_1_	*c*_2_	*c*_3_	*c*_4_	*c*_5_
0.419941	−0.968318	−1.740938	−1.766352	−0.664598

**Table 3:** Comparison on different polynomial arrangement.

Rule	*P*(*x*)	Required multiplier	Adders
Standard fourth order	Equation (1)	7 (4 fixed coefficients, 1 variable Coefficient, and 2 squarer)	4
Horner rule	Equation (4)	4 (1 fixed coefficient and 3 variable coefficients)	4
Reference [12]	Equation (5)	4 (2 fixed coefficients and 2 squarer)	4
Proposed rule	Equation (6)	3 (1 fixed coefficient, 1 squarer, and 1 variable coefficient)	5

**Table 4:** Quantized polynomial coefficients.

*a*_1,0_	*c*_1*q*_	*c*_2*q*_	*c*_3*q*_	*c*_4*q*_	*c*_5*q*_
1021/2^10^	3440/2^13^	−7932/2^13^	14262/2^13^	−14470/2^13^	−10889/2^14^

**Table 5:** Performance Comparisons with published work.

Reference	Design technique	Supply	SFDR (dBc)	Power (uW/MHz)	Clock (MHz)	Area (um^2^)	Normalized area ×10^5^	Process (nm)
[13]	Piecewise linear	3.3	84.2	NA	320	282000	23	350
[10]	Eighth-order polynomial	1.8	95	160	500	95000	29.3	180
[9]	Two segment fourth order	2.5	90	410	200	720000	115.2	250
This paper	Hybrid polynomial	0.9	90	6.55	430	908	5.67	40
